# Role of Percutaneous Liver Biopsy

**DOI:** 10.5812/hepatmon.854

**Published:** 2012-04-30

**Authors:** Mia Rodziewicz, Ricardo Moreno-Otero

**Affiliations:** 1 Department of Gastroenterology, CIBERehd, Madrid, Spain; 2 Health Research Institute Princess (IIS-IP). La Princesa University Hospital, Madrid, Spain

**Keywords:** Liver, Biopsy, Role

Dear Editor,

We read the interesting study by Szymczak et al. [1] regarding the safety and effectiveness of a blind percutaneous liver biopsy, where they concluded that the failure rate and the risk of complications are low if indications and contraindications are carefully considered. Moreover, these authors emphasize how important it is that biopsies are performed by skilled and experienced operators. This study raises controversy, however, as although a liver biopsy is reasonably safe, it is still an invasive procedure and it is unclear as to whether the histology results affect the management of patients with liver disease. In addition, the increasing availability of non-invasive techniques for staging hepatic fibrosis, together with new and advanced radiological, immunological, virological and molecular genetic tests, may undermine the future role of liver biopsy in clinical practice.

It has recently been reported (Björnsson E, et al., 2011) that patients with typical laboratory features of autoimmune hepatitis (AIH) rarely need a liver biopsy for their diagnosis and moreover, that most patients with hallmark features of AIH are likely to have a compatible liver histology. In the few patients with atypical histology, these findings also have little impact on their clinical management. Thus, a biopsy might be unnecessary in patients who meet the clinical criteria for AIH. This statement might be considered controversial, particularly in those patients who are difficult to categorize due to an atypical clinical presentation. Diagnosing drug-induced liver injury relies primarily on careful history taking; nevertheless, we had the opportunity to study two illustrative cases of patients with acute flares of severe hepatitis that underwent ultrasound-guided liver biopsy for diagnostic characterization. In these patients the histological findings played a decisive role in determining the specific therapeutic intervention required, which subsequently led to a remission of the acute disease [2][3].

It is generally accepted that a liver biopsy in patients with chronic hepatitis B is indicated in order to prescribe antiviral therapy, this is usually when serum ALT is very low and in suspected cases of advanced fibrosis (> F2) who are at risk of developing cirrhosis. Concerning chronic hepatitis C, a liver biopsy was previously recommended prior to commencing therapy in order to determine whether treatment was warranted and to assess disease prognosis (grading and staging). In current clinical practice this postulation has been revised and now the main role of a biopsy is the objective quantification of liver fibrosis into meaningful clinical groups, in particular, those at risk of progression to cirrhosis. Additionally, histological analysis in chronic viral hepatitis could tentatively include molecular analysis such as in situ hybridization or polymerase chain reaction used to detect occult viral infections, identification of premalignant lesions or in the context of clinical and research protocols [4][5].

In summary, Szymczak et al. [1] confirmed the safety and success of a blind liver biopsy and reference an extensive number of studies by experienced authors. In current medical practice, however, the importance of this invasive procedure is based on a well designed clinical rationale and knowledge of its precise indications. It can therefore be stated, that a decision on each individual case must be made in order to obtain helpful information for therapeutic interventions, and to establish an adequate follow-up protocol for prognostic control.

## References

[1] Szymczak A, Simon K, Inglot M, Gladysz A (2012). Safety and effectiveness of blind percutaneous liver biopsy: Analysis of 1412 procedures.. Hepat Mon.

[2] Moreno-Otero R, Garcia-Buey L, Garcia-Sanchez A, Trapero-Marugan M (2011). Autoimmune hepatitis after long-term methotrexate therapy for rheumatoid arthritis.. Curr Drug Saf.

[3] Moreno-Otero R, Trapero-Marugan M, Garcia-Buey L, Garcia-Sancchez A (2010). Drug-induced postinfantile giant cell hepatitis.. Hepatology.

[4] Garcia-Monzon C, Garcia-Buey L, Garcia-Sanchez A, Pajares JM, Moreno-Otero R (1993). Down-regulation of intercellular adhesion molecule 1 on hepatocytes in viral chronic hepatitis treated with interferon alfa-2b.. Gastroenterology.

[5] Sanz-Cameno P, Medina J, Garcia-Buey L, Garcia-Sanchez A, Borque MJ, Martin-Vilchez S (2002). Enhanced intrahepatic inducible nitric oxide synthase expression and nitrotyrosine accumulation in primary biliary cirrhosis and autoimmune hepatitis.. J Hepatol.

